# Anorexic Readiness Syndrome in Women Engaging in Body-Shaping Exercise

**DOI:** 10.3390/nu18020206

**Published:** 2026-01-08

**Authors:** Katarzyna Walicka-Cupryś, Agnieszka Pelc, Anna Wojtoń

**Affiliations:** 1 Institute of Physiotherapy, Faculty of Health Sciences and Psychology, Collegium Medicum, University of Rzeszów, St. Warzywna 1A, 35-310 Rzeszow, Poland; kwalicka@ur.edu.pl (K.W.-C.); aniawojton97@gmail.com (A.W.); 2 University Centre for Research and Development in Health Sciences, Warzywna 1A, 35-310 Rzeszow, Poland

**Keywords:** Anorexic Readiness Syndrome, eating disorders, body-shaping exercise, strength training, menstrual disorders

## Abstract

**Background:** Eating disorders are increasingly diagnosed in young women, particularly during adolescence. The recently described Anorexic Readiness Syndrome (ARS) is more common than full-blown anorexia. It has been identified in female athletes engaging in disciplines focusing on the aesthetics of the body, in women involved in recreational exercise and in those who are not physically active but strive to achieve the “perfect” figure. The study aimed to assess the severity and prevalence of ARS in women regularly engaging in body-shaping physical activity. **Methods:** The study included 659 women aged ≥ 14 years who engaged in regular body-shaping physical activity, provided informed consent to participate in the study (in the case of minors, also the consent of a parent or legal guardian), and had no diagnosed chronic diseases. The level of ARS was assessed using a questionnaire measuring attitudes toward food, supplemented with a specially designed survey consisting of 32 questions and a personal data form. Based on the frequency of body-shaping physical activity, participants were divided into two groups: the study group comprised women exercising ≥ 3 times per week (*n* = 301), while women exercising < 3 times per week constituted the control group (*n* = 358). The analyses examined the relationships between ARS, frequency of body-shaping physical activity, BMI, and menstrual irregularities. **Results:** Medium or high ARS level was identified in over 96% (*n* = 637) of the respondents. The level of ARS was significantly related to the allocation into the group (*p* = 0.034) and the weekly hours of physical activity (*p* = 0.011 in the control group; *p* = 0.020 in the study group). There was a correlation between ARS and menstrual irregularities (*p* = 0.001). Weak but significant correlations were identified for awareness of eating disorders (V = 0.20; *p* = 0.001), adherence to a special diet (V = 0.18; *p* < 0.001) and self-assessed health (V = 0.18; *p* < 0.001). **Conclusions:** Higher ARS levels were observed in women reporting greater weekly physical activity. No significant associations were found between ARS and body mass index or body weight. Medium and high ARS levels were significantly associated with self-reported menstrual disturbances, while most participants with elevated ARS were unaware of disordered eating risk.

## 1. Introduction

In an age when enormous attention is paid to one’s appearance, and comparisons are made with the beauty ideals promoted by the media, eating disorders have become an epidemic of the twenty first century [1]. This problem most often affects adolescent females who experience a decline in self-esteem [2], as a result of which they excessively engage in exercise [3] and follow a strict diet [4].

The eating disorders, which are still most commonly investigated, include anorexia and bulimia [5]. However, more and more attention is paid to subclinical abnormalities that do not meet the full diagnostic criteria but are manifested by disturbances in food intake, a distorted attitude towards one’s own body, or emotional, cognitive, and behavioural abnormalities. These phenomena were taken into account in the concept of Anorexic Readiness Syndrome (ARS) proposed by Ziółkowska [6]. ARS is understood as a set of early signs, primarily related to cognitive and behavioural functioning, that may be indicative of anorexic tendencies. Being a preventive measure, the construct makes it possible to identify early symptoms of abnormal eating behaviours and rigid, critical attitudes toward one’s own body in children and adolescents [7,8].

ARS is often observed in female athletes engaging in sports where the body is evaluated by coaches, referees, and the audience, and it significantly affects the final result during competitions [9]. An athlete who is dissatisfied with her appearance has significantly lower self-esteem, which also leads to a decline in efficiency, a loss of motivation for high-intensity training and for continued work on her body [10]. Driven by criticism from those around, the athlete strives to attain a perfect figure which gradually leads to eating disorders, overtraining, hormonal imbalances, chronic illnesses, as well as mental and social problems [11].

According to a number of studies, eating disorders are identified not only in women focusing on body-shaping strength training, but also in physically active women who do not engage in exercise aiming to improve their appearance [12,13]. What is more, this problem is also found in retired sportswomen, since maintaining their body shape is a way for them to confirm their earlier athletic identity [14].

It is common not only for female competitive athletes, but also for women engaging in recreational sports and those who do not exercise at all to have a critical view of their body. Furthermore, what many women share is the desire to have a perfect figure, which on the one hand motivates them for change and improvement, but on the other hand leads to a pathological desire to be perfect [15].

The aim of this study was to assess the severity and prevalence of ARS in women regularly involved in body-shaping exercise. The hypothesis was adopted that the level of ARS is related to the type and duration of physical activity, as well as to selected indicators of women’s health. Research focusing on this issue is particularly important because early symptoms of eating disorders are increasingly common and which may lead to serious consequences, both in physical and mental health. By understanding the prevalence of this problem in physically active women, we can more effectively identify the most at-risk groups and emphasise the importance of education and preventive measures.

## 2. Materials and Methods

### 2.1. Study Participants

The study involved 659 physically active women, aged 14–55 years, (x = 20.77 ± 4.13 SD), with BMI 21.44. The study group comprised 301 women (45.7%) engaging in body-shaping exercise, and the control group consisted of 358 women (54.3%) engaging in physical activity without a focus to body shaping. The statistical analyses took into account the groups distinguished this way. The analysis showed no significant differences between the groups in terms of age, height, weight, and BMI (Table 1).

### 2.2. Study Qualification

The study was carried out in a group of women engaging in body-shaping physical activity. For the purposes of analysis, the classification into study groups was based on the frequency of exercise. In line with the assumptions based on the literature [16,17,18], the body-shaping group comprised women who exercised three or more times a week. The women engaged in physical activity for an average of 5.29 h per week (Me = 5.5, SD = 2.94).

The women who exercised less than three times a week were included in the control group. The study included women aged ≥ 14 years who engaged in regular physical activity, gave their informed consent to participate in the study (in the case of minors, also the consent of a parent or legal guardian) and had no diagnosed chronic diseases. Exclusion criteria included self-reported pregnancy, psychiatric disorders, endocrinological conditions, cardiovascular or metabolic diseases. Participants who did not meet the above criteria were excluded from the study.

After qualifying for the study, the participants were divided into two groups depending on the frequency of physical activity aimed at shaping the body: the study group consisted of women exercising ≥ 3 times a week (*n* = 301), while the control group consisted of women exercising < 3 times a week (*n* = 358).

### 2.3. Questionnaire

The survey was disseminated online via social media platforms and internet groups dedicated to physically active women and individuals interested in physical activity and health-related topics. The materials used in the present study included an online survey which consisted of two parts. The first part comprised a diagnostic screening questionnaire developed by Ziółkowska et al. [2] and administered in its original Polish-language version, aimed at identifying young women exhibiting abnormalities in their attitudes toward food and body image. The statements addressed weight loss, attitudes towards physical attractiveness, attitudes towards food, as well as family relationships and aspects of upbringing. High scores on the questionnaire are indicative of ARS, and low scores show there is no such problem. The questionnaire consisted of 20 statements with Yes or No responses. The original Polish-language version of the questionnaire was used, as developed by the author and previously applied in Polish populations for screening purposes. The ARS questionnaire is a screening instrument developed by Ziółkowska to identify early cognitive and behavioural features associated with anorexic tendencies. The tool has demonstrated acceptable validity and reliability in previous studies conducted in Polish adolescent and young adult populations and is intended to assess risk rather than to establish a clinical diagnosis.

The level of ARS, assessed on a scale corresponding to the number of positive responses, is defined as follows:

0–6 (low ARS level)—low risk of developing a disease linked to eating disorders;

7–13 (medium ARS level)—moderate risk of developing a disease linked to eating disorders;

14–20 (high ARS level)—high risk of developing a disease linked to eating disorders.

The original cut-off points proposed by the author were applied, and no sensitivity analyses using alternative thresholds were conducted.

The ARS scale was originally developed for younger age groups, but given that attitudes towards appearance are not limited to adolescence and persist into adulthood, the scale was applied to the entire sample.

The second part of the survey covered three issues, i.e., exercise, health status and dietary approach. It also included a personal details form with basic questions about the characteristics of the respondent. The second part of the survey was an author-designed questionnaire and was not formally validated; it was used to collect descriptive self-reported data. Based on the results of the first part of the survey, the respondents were divided into groups according to the ARS index. Menstrual disorders were assessed based on self-reported responses to survey questions regarding irregular, absent, or otherwise disturbed menstrual cycles and were not clinically verified.

Subsequently, a comparative analysis of the groups was performed taking into account the information acquired in the second part of the survey with regard to the weekly number of training sessions and hours dedicated to physical activity, adherence to a strictly defined diet, assessment of health, menstrual disorders and the women’s awareness of their ARS diagnosis.

Potential excessive weight fluctuations were identified by calculating the difference between the highest and the lowest body weight reported by the respondent during the period in which she began to consciously manage her body shape and engage in regular physical activity. A difference of ≥8 kg was considered an excessive fluctuation.

### 2.4. Statistical Analysis

The statistical analyses were computed using Statistica 13.3. Differences between two independent groups were assessed using the Mann–Whitney U test, whereas differences among more than two independent groups were assessed using the Kruskal–Wallis H test (a non-parametric equivalent of one-way ANOVA), with the test statistic expressed as a chi-square (χ^2^) value. Non-parametric tests were chosen due to the ordinal nature of the ARS measure, unequal group sizes, and the lack of normal distribution of selected continuous variables. Relationships between categorical variables were examined using the chi-square test, and the strength of associations between nominal variables was determined using Cramér’s V, interpreted as follows:

V = 0.00–0.30—weak;

V = 0.31–0.50—moderate;

V = 0.51–0.70—strong;

V = 0.71–1.00—very strong.

The level of statistical significance was defined as *p* < 0.05.

### 2.5. Ethics

The study was conducted in accordance with the Declaration of Helsinki, and approved by the Bioethics Committee of Rzeszow University, approval number 5/112014 dated 26 November 2014.

## 3. Results

The findings show that the largest number of respondents (396 women; 60.09%) had medium ARS level. High ARS level was identified in 241 women (36.57%), and low ARS level in 22 participants (3.34%) only. The classification of respondents according to their ARS score in both groups is presented in Table 2. The analysis shows a significant relationship between ARS level and allocation into the group (Cramér’s V = 0.1; *p* = 0.034). The acquired data can be used to present this relationship without the distinction into the specific groups.

Analyses were also conducted to assess the differences in BMI, body weight and hours dedicated to strength training relative to the ARS level. There were no significant differences in BMI in both the control group, χ^2^ = 4.287; *p* = 0.117, and the study group, χ^2^ = 2.883; *p* = 0.237. Likewise, the findings showed no significant differences in body weight relative to the ARS level in the control group, χ^2^ = 0.922; *p* = 0.631, and in the study group, χ^2^ = 2.806; *p* = 0.246. Only in the case of weekly hours of strength training the findings showed significant differences between ARS levels both in the control group, χ^2^(2) = 9.019; *p* = 0.011, and in the study group, χ^2^ = 7.823; *p* = 0.020 (Table 3).

In order to examine more closely the differences in the number of weekly hours of physical training relative to ARS level, an analysis was carried out in both groups. In the control group the women with high ARS (Me = 4.38; SD = 2.63) reported significantly more weekly hours of physical training than the women with medium ARS (Me = 3.74; SD = 2.37), U = 11,198.500; *p* = 0.019, and those with low ARS level (Me = 2.60; SD = 1.27), U = 340.000; *p* = 0.020. Similarly, in the study group the respondents with high ARS (Me = 6.98; SD = 3.09) also dedicated significantly more hours to physical training per week compared to the respondents with medium ARS (Me = 6.50; SD = 2.72), U = 8692.500; *p* = 0.007. The remaining comparisons showed no statistical significance (Table 4).

Analysis of the responses to questions concerning self-rated health, adherence to a strict diet, existence of eating disorders and body weight fluctuations showed significant, albeit weak, correlations with ARS levels in the first three variables. The level of ARS was significantly related to self-assessed health status (Cramér’s V = 0.18; *p* < 0.001), adherence to a diet (Cramér’s V = 0.18; *p* < 0.001) as well as existence of issues linked to eating disorders (Cramér’s V = 0.20; *p* < 0.001). In the case of body weight fluctuations no significant correlations were found in any ARS group: Low ARS (Cramér’s V = −0.02; *p* = 0.361), Medium ARS (Cramér’s V = 0.03; *p* = 0.597) and High ARS (Cramér’s V = −0.03; *p* = 0.657) (Table 5).

Overall, menstrual disorders were reported by 375 women, including 172 women in the study group and 203 women in the control group. The findings show that menstrual disorders are significantly related to ARS levels, χ^2^ = 31.964; *p* = 0.001. Women with moderate and high ARS levels reported menstrual disorders significantly more often than women with low ARS levels (Table 6).

## 4. Discussion

Today ARS is a more and more common problem, particularly observed in adolescent females [10,11]. Zięba et al. [19] reported that girls were more likely than boys to reduce food intake, show emotional eating, and attempt to lose weight, while boys were less susceptible to emotions and the impact of social media [19]. ARS frequently leads to eating disorders which are among the major health issues identified in young women in the twenty first century. The cult of thinness promotes restrictive dieting and excessive physical exercise, especially among female athletes [4]. In the current study, over 96% of the respondents were classified as having medium or high ARS levels. A high ARS level was identified in over 36% of the sample, which is higher than proportions reported in several earlier studies [9,20,21]. However, as the applied questionnaire is a screening tool, these proportions should be interpreted as indicating elevated risk rather than confirmed pathology. Women reporting higher training frequency were classified with medium or high ARS approximately 10% more often than women in the control group; however, training frequency should not be equated with aesthetic or body-shaping motivation, as such motivation was not assessed directly. Only 3.34% of respondents were classified as having a low ARS level, indicating a lower risk within this subgroup. Although several associations reached statistical significance, the corresponding effect sizes (Cramér’s V = 0.10–0.20) indicate weak relationships and should therefore be interpreted with caution. Nevertheless, these findings suggest modest associations that may still be relevant at the population level.

A study by Ołpińska-Lischka [1] involving female dancers from Poland and Germany established that ARS was more common in younger women (<25 years), with high levels identified more often in Polish and moderate levels prevailing in German dancers. Similar findings were reported by Chalcarz [21] who investigated female students of tourism and recreation; in this case the problem was identified at a significantly higher rate in the age group of 18–25 years, compared to older respondents [21]. Although previous studies have reported age-related differences in ARS, age-group comparisons were not performed in the present study due to the skewed age distribution of the sample and the screening nature of the applied instrument.

The analysis showed that ARS level increased with the duration of exercise. Women who paid attention to their figure exercised more, engaged in strength training more often (≥3 times a week) and had longer history of training. Similar results were reported by Chalcarz [20,21] who found that female students of middle schools following special programmes in sports on average engaged in exercise three times more than students following general education programmes although high ARS level in this case was not associated with longer duration of training. Most commonly they had continued training for 1–3 years; female sports students with low and moderate level had a longer history of training (3–5 years). In a group of female students of Physical Education Academy the highest percentage of high and moderate ARS level was found in those with a history of training for 1–5 years and then 5–10 years. A similar trend was identified in female judo athletes–ARS was more severe after more than 5 years of training [22].

Women who engaged in strength training three-five times per week for more than one year reported such goals as improved fitness, weight loss, silhouette modelling, body toning and increase in strength and muscle mass [23]. Pursuit of these goals, especially if it is motivated by dissatisfaction with one’s body may lead to decline in one’s mental well-being, including an increased risk of depression and mood disorder [24]. A study showed that anorexia readiness syndrome was associated with excessive physical activity and disturbed eating habits manifested in strict diets, fasting or efforts to get rid of food consumed earlier [2]. As many as 49.07% of the respondents were at the time following a diet, 30.71% had done that in the past, and only 20.21% had never followed a strict diet, mainly the women with low ARS level. The respondents with medium and high ARS level most commonly followed diets defined based on their own knowledge. Similar trends were identified by researchers investigating female students of middle schools [20], female university students [21] and female judo athletes [22]; in these studies respondents with high ARS level reported strict diets, fasting, and restrictions on fats and carbohydrates [20,21,22].

Furthermore, most of the middle school and university students investigated were aware of the energy values of products and felt remorse after overeating. As the ARS level decreased, the percentage of such behaviours also decreased. Similar findings were shown in dancers from Poland and Germany [1], in this case however the women more commonly experienced dietary problems, and that was also reported by Łebek et al. [25] who investigated females aged 15–18 years. Tuszyńska-Bogucka [26] showed that over 60% of young women (14–26 years) were dissatisfied with their appearance, but only 4.75% resorted to fasting or rigorous diets [26]. This is far less than in other studies [1,11,20,21,22] and in the present study.

Excessively rapid weight loss may be associated with chronic diseases, full-blown anorexia or other eating disorders [27]. The current study included analysis of weight fluctuations; the respondents reported their highest and lowest body weight since they started to focus on their figure and physical activity. The greatest fluctuations were observed in women with medium and high ARS levels. In the body-shaping group excessive loss of body weight may be linked to such factors as preparations for sporting competitions or increased attention to appearance [10,11]. A desire to attain lower body weight is common among physically active women and professional athletes, and in physically inactive women, as reported by Chytra-Gędek et al. [28]. A study conducted among online communities associated with pro-anorexia content among young women showed that nearly 60% of respondents desired to reduce their body weight, although in most cases their BMI was below normal [29].

Menstrual disorders in female athletes and women engaging in recreational physical activity are increasingly common. This is linked to the aforementioned excessive physical activity and caloric deficit, as well as macronutrient deficiency resulting from strict diets [30,31].

The present study did not identify any significant differences in the prevalence of menstrual disorders between the groups, but significant correlations were found in women with medium and high ARS levels. More than half of the respondents reported menstrual disorders, whereas those with low ARS level reported this problem at a rate of only 1.64%. The results clearly show a relationship between ARS levels and the menstrual cycle. For comparison, menstrual disorders were identified at a rate of 23% in female cross-country skiers [30] and in 27% of endurance athletes who also presented higher ARS levels [31].

Research conducted in a group of female acrobatic gymnasts has shown that early diagnosis of eating disorder symptoms and the related preventive measures are crucial for health and sporting performance [32]. This suggests that similar measures are also needed for women engaging in body-shaping exercise since in this case the pressure to look good and the specific aesthetic standards may promote unhealthy eating habits. Nutrition education and psychological support provided to this group could bring similar benefits to those observed in the gymnasts.

## 5. Limitations

The present study has several limitations that should be considered when interpreting the results. The use of a self-report online survey is inherently associated with subjectivity and potential over- or under-reporting, particularly with respect to health-related variables such as menstrual disturbances, dietary practices, and body weight history, which were not clinically verified. The interpretation of ARS prevalence therefore requires particular caution. The applied questionnaire functions as a screening tool rather than a diagnostic instrument, which may contribute to an overestimation of medium and high ARS levels. In addition, the psychometric properties of the questionnaire, including internal consistency and sensitivity to alternative cut-off points, were not re-evaluated in the present sample, which should be taken into account when interpreting the findings. Moreover, the wide age range of participants may limit the comparability of ARS prevalence estimates across age groups, as the questionnaire was primarily developed with younger populations in mind. Furthermore, the use of convenience sampling and recruitment among physically active women may have introduced selection bias, favouring individuals more engaged with body- and health-related issues, which could partly explain the high proportion of respondents classified as having elevated ARS. Additionally, the use of bivariate analyses did not allow for simultaneous adjustment for multiple covariates; however, this analytical approach was deliberately chosen to preserve transparency and clinical interpretability of individual associations. Finally, the cross-sectional and observational design of the study precludes causal inference and limits conclusions regarding the directionality of associations between physical activity patterns, weight fluctuations, and ARS. The operational criteria applied for certain variables should therefore be interpreted as indicative rather than definitive. Future research should focus on more homogeneous age groups and incorporate additional sociodemographic variables, such as income level and family background, which may influence dietary behaviours and health outcomes.

## 6. Conclusions

1. Higher levels of ARS were observed in women reporting greater weekly duration of physical activity.
2. The association between ARS level and participation in body-shaping physical activity was weak and should be interpreted with caution.
3. No significant associations were found between ARS level and body mass index, body weight, or body weight fluctuations.
4. Medium and high ARS levels were significantly associated with self-reported menstrual disturbances.
5. Most participants with elevated ARS levels did not report a diagnosed or suspected eating disorder, indicating limited awareness of early disordered eating patterns.

## Figures and Tables

**Table 1:** Anthropometric characteristics in both groups.

	Group	N	$\overset{-}{x}$	Me	Min.	Max.	SD	t	*p*
**Age [years]**	Study	301	21.09	21.00	14.00	45.00	4.20	−1.787	0.223
	Control	358	20.46	20.00	14.00	55.00	4.07		
**Height [cm]**	Study	301	166.70	166.00	150.00	184.00	6.16	1.074	0.324
	Control	358	167.23	167.50	150.00	186.00	6.29		
**Body weight [kg]**	Study	301	60.00	60.00	37.00	98.00	4.72	0.477	0.520
	Control	358	60.08	59.00	37.00	110.00	9.86		
**BMI [kg/m^2^]**	Study	301	21.56	21.30	14.82	34.72	2.44	−0.033	0.979
	Control	358	21.44	21.13	14.82	34.77	3.01		

N—number of observations; $\overset{-}{x}$—mean; Me—Median; Min.—minimum value; Max.—maximum value; SD—standard deviation; t—Student’s *t*-test; *p*—test probability value.

**Table 2:** The ARS level in the two groups.

Group	Level of ARS						
	Low		Medium		High		
	N	%	N	%	N	%	
**Study**	10	3.32	165	54.82	126	41.86	χ^2^ = 6.757 ***p* = 0.034**
**Control**	12	3.35	231	64.53	115	32.12	
**Total**	22	3.34	396	60.09	241	36.57	

Percentages are calculated relative to the number of participants in each group; percentages in the Total row refer to the entire sample. N—number of observations; χ^2^—chi-square test; *p*—probability value; bold—statistical significance at *p* < 0.05.

**Table 3:** Relationship between the specific group as well as ARS level and the variables: BMI, body weight and weekly hours of physical training.

			Level of ARS			*df*	*χ* ^2^	*p*
			Low	Medium	High			
**Control Group**	**BMI [kg/m^2^]**	**N**	10	227	112	2	4.287	0.117
		**Me**	21.63	21.32	21.20			
		**SD**	2.52	3.32	4.60			
	**Body weight [kg]**	**N**	10	227	112	2	0.922	0.631
		**Me**	61.10	60.10	59.79			
		**SD**	10.51	10.79	12.59			
	**Weekly training time**	**N**	10	227	112	2	9.019	**0.011**
		**Me**	2.60	3.74	4.38			
		**SD**	1.27	2.37	2.63			
**Study Group**	**BMI [kg/m^2^]**	**N**	10	163	126	2	2.883	0.237
		**Me**	21.67	21.84	21.22			
		**SD**	2.28	2.67	2.11			
	**Body weight [kg]**	**N**	10	163	126	2	2.806	0.246
		**Me**	61.10	60.86	58.90			
		**SD**	6.42	8.82	7.12			
	**Weekly training time**	**N**	10	163	126	2	7.823	**0.020**
		**Me**	5.60	6.50	6.98			
		**SD**	2.63	2.72	3.09			

N—Number of observations; Me—Median; SD—standard deviation; *df*—Degrees of freedom; χ^2^—Kruskal–Wallis test statistic; *p*—probability value; bold—statistical significance at *p* < 0.05.

**Table 4:** Weekly hours of physical training relative to the ARS levels in both groups.

		N	Me	SD	U	*p*
**Control Group**	**Low ARS level**	10	2.60	1.27	836.000	0.105
	**Medium ARS level**	228	3.74	2.37		
	**Low ARS level**	10	2.60	1.27	340.000	**0.020**
	**High ARS level**	114	4.38	2.63		
	**Medium ARS level**	228	3.74	2.37	11,198.500	**0.019**
	**High ARS level**	114	4.38	2.63		
**Study Group**	**Low ARS level**	10	5.60	2.63	771.500	0.695
	**Medium ARS level**	165	6.50	2.72		
	**Low ARS level**	10	5.60	2.63	484.500	0.186
	**High ARS level**	126	6.98	3.09		
	**Medium ARS level**	165	6.50	2.72	8692.500	**0.007**
	**High ARS level**	126	6.98	3.09		

N—number of observations; Me—Median; SD—standard deviation; U—the test statistic for the Mann–Whitney U Test; *p*—probability value; bold—statistical significance at *p* < 0.05.

**Table 5:** ARS level in relation to health status, adherence to a diet, and eating disorders.

	Group	Would You Say You Are in Good Health?							
		Definitely Not	Probably Not	Hard to Say	Probably Yes	Definitely Yes	Total	V	*p*
**Low ARS level**	**Study**	0	0	3	5	2	10	0.18	**<0.001**
	**Control**	0	0	5	5	2	10		
**Medium ARS level**	**Study**	8	21	61	117	21	228		
	**Control**	3	17	31	86	28	163		
**High ARS level**	**Study**	2	13	40	47	11	113		
	**Control**	2	14	39	7	7	126		
**Total**		15	65	179	267	71	648		
	**Group**	**Do You Follow a Strict Diet?**							
		**Never**	**Not Now, I Did in the Past**	**Yes, Self-Prepared**	**Yes, by a Dietitian/Trainer**	**Yes, by a Friend**	**Total**	**V**	***p***
**Low ARS level**	**Study**	8	1	0	1	0	10	0.18	**<0.001**
	**Control**	3	4	2	0	1	10		
**Medium ARS level**	**Study**	63	84	76	5	0	228		
	**Control**	30	34	81	14	4	163		
**High ARS level**	**Study**	17	40	53	2	1	113		
	**Control**	10	36	68	11	1	126		
**Total**		131	199	280	33	5	648		
	**Group Type**	**Have You Been Diagnosed with or Suspected of Having an Eating Disorder?**							
		**Not Diagnosed/** **Suspected**		**Diagnosed/** **Suspected**		**Total**		**V**	***p***
**Low ARS level**	**Study**	10		0		10		0.20	**<0.001**
	**Control**	8		2		10			
**Medium ARS level**	**Study**	129		35		164			
	**Control**	177		49		226			
**High ARS level**	**Study**	81		44		125			
	**Control**	67		46		114			
**Total**		472		176		648			
	**Group Type**	**Body Weight Fluctuation Level**							
		**Small Fluctuations**		**Excessive** **Fluctuations**		**Total**		**V**	***p***
**Low ARS level**	**Study**	5		5		10		−0.2	0.361
	**Control**	7		3		10			
**Medium ARS level**	**Study**	73		151		224		0.03	0.597
	**Control**	49		114		163			
**High ARS level**	**Study**	27		86		113		−0.03	0.657
	**Control**	33		92		125			

V—Cramér’s V; *p*—probability value; bold—statistical significance at *p* < 0.05.

**Table 6:** ARS levels in relation to menstrual disorders in both groups.

	Study Group		Control Group		χ^2^	*p*
	N	%	N	%		
**Low ARS level**	2	1.16	4	1.97	31.964	**0.001**
**Medium ARS level**	84	48.84	109	53.69		
**High ARS level**	86	50.00	90	44.34		
**Total**	172	100.00	203	100.00		

N—number of observations; χ^2^—chi-square test statistic of the Kruskal–Wallis test; *p*—probability value; bold—statistical significance at *p* < 0.05.

## Data Availability

The original contributions presented in this study are included in the article. Further inquiries can be directed to the corresponding author.

## Abbreviations

The following abbreviation is used in this manuscript:

ARS	Anorexic Readiness Syndrome
